# Chimpanzees Extract Social Information from Agonistic Screams

**DOI:** 10.1371/journal.pone.0011473

**Published:** 2010-07-14

**Authors:** Katie E. Slocombe, Tanja Kaller, Josep Call, Klaus Zuberbühler

**Affiliations:** 1 Department of Psychology, University of York, York, United Kingdom; 2 Department of Developmental and Comparative Psychology, Max Planck Institute for Evolutionary Anthropology, Leipzig, Germany; 3 School of Psychology, University of St Andrews, St Andrews, United Kingdom; University of Sussex, United Kingdom

## Abstract

Chimpanzee (*Pan troglodytes*) agonistic screams are graded vocal signals that are produced in a context-specific manner. Screams given by aggressors and victims can be discriminated based on their acoustic structure but the mechanisms of listener comprehension of these calls are currently unknown. In this study, we show that chimpanzees extract social information from these vocal signals that, combined with their more general social knowledge, enables them to understand the nature of out-of-sight social interactions. In playback experiments, we broadcast congruent and incongruent sequences of agonistic calls and monitored the response of bystanders. Congruent sequences were in accordance with existing social dominance relations; incongruent ones violated them. Subjects looked significantly longer at incongruent sequences, despite them being acoustically less salient (fewer call types from fewer individuals) than congruent ones. We concluded that chimpanzees categorised an apparently simple acoustic signal into victim and aggressor screams and used pragmatics to form inferences about third-party interactions they could not see.

## Introduction

Theories of the origins of human language draw heavily on comparative evidence of extant primates [e.g. 1], [2]. To date, vocal research has focussed almost exclusively on monkey species whereas gestural research relies almost entirely on apes [3]. It is imperative that research investigating vocal competencies in apes is forthcoming to provide a valid comparison and also to test whether the monkey evidence is a case of convergent evolution. So far, only a small number of studies have tested call comprehension in great apes [4], [5] and thus we know very little about the abilities of great apes to extract and integrate information from calls; a vital part of human communicative abilities.

We address this issue with a study on how chimpanzees process vocal utterances produced during agonistic interactions. Many species of animal produce vocalisations during agonistic interactions, in the roles of both the aggressor and victim. Specific calls are produced to defend a territory (sand gobies [6]; Lusitanian toadfish [7]; crickets [8]) or a food source (pipistrelle bats [9]; dogs [10]) and these signals tend to be honest signals of body size and therefore fighting ability of the caller (dogs [11]; sand gobies [6]; crickets [8]; red deer [12]). Many species also produce specific vocalizations when experiencing aggression, including female rats [13], rhesus monkeys [14] and chimpanzees [15].

Detailed investigations of signalling during agonistic contexts have revealed advanced social and communication skills in some monkey species, with rhesus monkeys (Macaca *mulatta*) being capable of making inferences about an ongoing conflict based on the victim screams of their offspring [14]. In chimpanzees, observational studies of agonistic encounters have already revealed context-specific vocal behaviour in a number of ways. Firstly, chimpanzee victims vary the acoustic structure of their screams as a function of the severity of aggression they are facing [16], and field experiments have shown that receivers can discriminate between these differences [5]. Secondly, chimpanzees also vary their screams depending on their social role in an agonistic interaction with victims and aggressors producing acoustically distinct screams [15], but it is currently not known whether others attend to these acoustic differences.

Here, we tested whether, as bystanders of social conflicts, chimpanzees are able to extract information about the social role (victim or aggressor) of the two protagonists from their screams and integrate this with more general social knowledge to make inferences about the nature of a fight they cannot see. This is an ecologically valid problem for chimpanzees who typically dwell in dense forested habitat [17]. Low visibility and a fission-fusion social system have the effect that chimpanzees typically hear many more fights than they see (Slocombe, unpublished data), potentially favouring the evolution of advanced comprehension abilities.

To test whether chimpanzee listeners were able to extract social information from agonistic screams, we presented adult individuals with congruent and incongruent sequences of screams, that is, stimuli that did or did not violate the existing social hierarchy. We adapted the basic experimental design, originally used by Cheney and colleagues [18] to examine causal reasoning in baboons, to address a different question of call comprehension in chimpanzees. We expected individuals to show greater orienting responses to incongruent than congruent stimulus sequences, in line with other animal and human infant research [e.g. 19]. Incongruent sequences consisted of low-ranking aggressor screams followed by high-ranking victim screams. These sequences represented an interaction that was at odds with the existing social hierarchy. Congruent stimuli consisted of exactly the same scream sequences, but with the addition of a pant hoot vocalisation from a displaying male who outranked both screaming individuals. Congruent call sequences thus simulated a plausible situation where it was most likely that the high-ranking's victim scream was elicited by the dominant male's intimidation display, rather than the low-ranking individual's social machinations. Individual recognition was necessary to infer the congruency of the sequences and there is good evidence that chimpanzee calls are individually distinctive vocalisations that can also be discriminated by others [e.g. 20], [21].

If chimpanzees are able to discriminate and understand the meaning of the different calls, as well as the social constraints under which the two callers operate, they should look longer at the speaker after hearing incongruent sequences than congruent ones. In contrast, if chimpanzees are unable to do so, their response pattern should be random, or in the other direction since congruent sequences are acoustically more salient than the incongruent ones.

## Results

We compared the looking duration of subjects in response to the two playback sequences. As a group, subjects looked significantly longer at the speaker in the incongruent compared to the congruent condition (incongruent: median 7.13s, inter-quartile range 10.29; congruent: median 4.13 s, inter-quartile range 6.35; Wilcoxon test Z = -2.31, p = 0.02; table 1).

**Table 1:** Duration subjects looked towards the speaker in the minute after the onset of the victim screams, which were the crucial part of the incongruent or congruent call sequences.

Subject	Looking duration to speaker (s)	
	Incongruent	Congruent
Riet	4.97	2.50
Sandra	28.26	14.58
Fraukje	29.90	1.96
Corry	5.24	3.40
Ulla	1.04	0.75
Frodo	7.90	7.90
Robert	6.36	5.24
Patrick	4.24	1.80
Swela	10.68	4.86
Dorein	8.04	9.40

At the individual level, one subject showed equal responses to both trial types. The nine remaining individuals discriminated the two sequences with 8 out of 9 looking longer at the incongruent than the congruent stimuli, with only one individual showing the reverse pattern, a level significantly above that expected by chance (Binomial (0.5), exact p = 0.039).

To control for potential variation in the amount of natural orientation subjects displayed towards the sleeping room, we examined the looking duration to the speaker in the minute after the victim scream, once the baseline looking time at the speaker in the minute before playback had been subtracted. Subjects still looked longer at the speaker in response to the incongruent than the congruent stimuli (median 5.80 s, inter-quartile range 8.84; median 0.56 s, inter-quartile range 7.94); Wilcoxon test Z = -2.80, p = 0.002.

No differences were found in terms of locomotor behaviour. The incongruent playbacks elicited four of nine individuals to approach the doors to the sleeping room, the congruent playbacks three of nine.

## Discussion

We have demonstrated with this study that chimpanzees showed greater orientation responses to incongruent than congruent scream sequences, suggesting that they extracted information about the social role of the two callers from their agonistic screams and made sense of the simulated interaction by interpreting their vocal behaviour within a wider social context. Specifically, subjects responded stronger to sequences that simulated a social interaction that violated the dominance hierarchy, compared to sequences that did not. To perceive the underlying social anomaly subjects could not simply rely on the acoustic surface features of the stimuli, but must have been able to make some inferences about the direction of aggression by assigning two distinct social roles to the callers (victim and aggressor). Results further demonstrate that chimpanzees can identify individual group members from their agonistic screams and that they can integrate invisible events simulated by the calls with their existing social knowledge about the expected social interactions of the call producers with regards to their social standing in the group.

The observed differences in looking responses cannot be explained by the acoustic features of the playback stimuli. Subjects responded comparatively weakly to congruent stimuli despite these sequences being more salient and attention-grabbing in a number of ways. Congruent stimuli were acoustically more salient than the incongruent ones, as they contained more call types from more individuals. In addition, the congruent sequence simulated interactions involving more individuals, including a top-ranking male, which generally evokes much interest in naturally occurring fights (Slocombe, unpublished data). Despite these factors subjects responded relatively weakly to these sequences. It was also not the case that chimpanzees' behaviour could be explained in terms of a stronger response to novel sequences of calls, because the pattern of screaming in both conditions was identical, therefore equally novel.

This study has shown that chimpanzees are capable of extracting a range of social information from the calls of familiar group members. Given the wealth of studies showing that monkeys are very adept at extracting social information from calls [e.g.14], [ 18], [ 22] the current study indicates that such comprehension skills are also present in the great apes and as such phylogenetically old abilities. Further research into chimpanzee vocal behaviour may provide more evidence for continuity between elements of monkey and human vocal communication with considerable relevance for theories of language evolution.

In conclusion, in this study listeners meaningfully distinguished between victim and aggressor screams and integrated this information with pragmatic social knowledge in order to draw inferences about the nature of an interaction they could not see. Victim and aggressor screams are two distinct scream types within the largely graded vocal system of chimpanzees, which are discriminated by receivers and appear to be meaningful to them. This result further demonstrates that even in graded vocal systems meaningful acoustic variation can be present within the major call types, which, combined with the ability to integrate pragmatic knowledge, enables individuals to communicate in complex ways.

## Materials and Methods

### Subjects

3 male and 7 female chimpanzees (10-31 years old), housed at the Wolfgang Köhler Primate Research Centre (WKPRC), Leipzig, Germany, participated in this study. The subjects were housed socially in a group consisting of 18 individuals in spacious, naturally designed indoor (430 m2) and outdoor (4000 m2) enclosures, and a sleeping room which consisted of five interlinked cages (each 5.1-7.3 m2). During the period of testing, all apes received their complete daily diet consisting of various fresh fruits, vegetables, leaves, cereals, eggs and meat, and were never deprived of food or water at any time. In addition to 4-5 feeds each day the apes had access to several enrichment devices from which they could extract nuts and fruits with the help of tools.

We determined the dominance relations between stimulus providers by analysing the patterns of pant-grunting behaviour, the standard indicator of rank relations in chimpanzees [17]. Pant-grunt data were collected on an all-occurrence basis during regular 2-hour observation sessions (4-5/week) at WKPRC from April-September 2007, which revealed the dominance relationships shown in table 2.

**Table 2:** Relative social dominance of all individuals serving as stimulus providers.

	Aggressor scream		Victim scream		Pant hoot	
Stimulus set	ID	Respect value†	ID	Respect value	ID	Respect value
1	Swela (11)	0.02	Riet (29)	0.16	Robert* (31)	86.60
2	Dorien (26)	0.03	Riet (29)	0.16	Frodo* (13)	135.00
3	Pia (9)	0.01	Dorien (26)	0.03	Frodo* (13)	135.00
4	Natascha (27)	0.13	Robert* (31)	86.60	Frodo* (13)	135.00

Parentheses: age in years; † Respect value  =  N pant grunts received +1/N pant grunts given +1; see [23].*  =  male.

### Ethical statement

The School of Psychology Ethics committee, University of St Andrews gave ethical clearance for this non invasive, behavioural study and, in accordance with ethical guidelines, we terminated any trial in which the subject became acutely distressed, for instance if producing a screaming tantrum. This only occurred once and the subject was immediately released to rejoin her group, before the playback occurred.

### Stimuli

The incongruent stimuli consisted of an aggressor scream bout of a lower-ranking individual followed directly by a victim scream bout of a higher-ranking individual. This sequence simulated an unusual event, because chimpanzees are rarely injured or made fearful by lower-ranking group members.

Congruent stimuli could have consisted of an inverted sequence (high-ranking aggressor scream then low-ranking victim scream). However, this approach would have introduced the confound of novelty when comparing the congruent and incongruent stimuli, as it is comparatively rare for a high-ranking individual to produce victim screams. In this case meaningful interpretation of a longer orientation to the incongruent stimuli (the only sequence to contain this call) would have been impossible as it could have reflected interest in (1) an unusual agonistic interaction that was only possible to understand with extraction of social information from the calls or (2) a relatively novel sound. In order to exclude this confound it was therefore imperative that the sequences of aggressor and victim screams remained identical in the two conditions.

**Figure 1:**
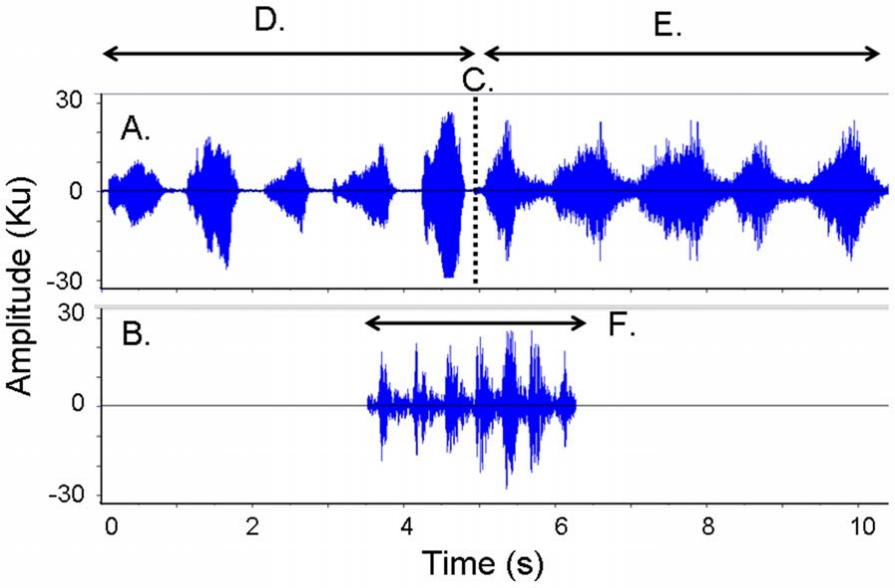
Waveform illustrations of an exemplar congruent stimulus. Illustrated are (A) Channel 1, (B) Channel 2, (C) The point where the aggressor scream ends and the victim scream starts, (D) Aggressor scream given by low ranking individual, (E) Victim scream given by high ranking individual, (F) Pant hoot build up given by dominant male. The incongruent stimuli consisted of Channel 1 only.

In order to construct the congruent sequence we thus kept the scream sequences identical and, in line with the original study by Cheney and colleagues [18], introduced calls from a third individual to make the sequence congruent. In this study we overlaid a pant-hoot build up given by a top-ranking male in the middle of the playback stimulus so it overlapped with parts of the aggressor (mean 1.48 sec) and the victim (mean 1.41 sec) calls (Fig. 1).

This way, the incongruent sequence became congruent with the dominance hierarchy in the group, due to the simulated presence of a third individual dominant to both the victim and aggressor. This sequence thus simulated two independent events, none of which violated any social expectations. Firstly, a low ranking individual attacking an unknown (silent) individual, and second a high ranking individual responding with victim screams to the male's display pant hoots, a common response to such intimidation displays [16].

Screams and pant hoots were recorded opportunistically during natural agonistic encounters in the group (June-August 2006 and June 2007). Only recordings made during unambiguous contexts were included (aggressor screams: physical contact, charging or lunging at victim; victim screams: physical contact or being chased by aggressor; pant hoots: display charges towards other group members). Acoustic analyses confirmed that the structure of these screams mirrored the acoustic structure of victim and aggressor calls reported for wild chimpanzees [15; see supplementary methods in file S1].

All calls were recorded using a SENNHEISER K6/ME67 directional microphone and a MARANTZ PMD660 solid state recorder (sampling rate of 44.1 kHz, 16 bits accuracy). Calls were edited using RAVEN Pro 1.3. Stimuli were stored as .wav files on a Toshiba Tecra-M3 laptop and broadcast through a NAGRA-DSM speaker.

Stimulus scream bouts contained 5 or 6 calls, had a mean duration of 4.87 sec, (SD = 0.64), and were clear from other chimpanzees calling and excessive background noise. The screams were approximately equalized in amplitude (Mean RMS volume = 5212, SD = 986). An average of 3.1 sec (SD  = 0.1) of the build up of the pant hoot was used. The playback volume for each stimulus set was modified so it sounded natural to the experimenter from the location the subject would hear it from. The incongruent and congruent stimulus pairs were identical in total duration and were played at identical amplitudes. Four different stimulus sets were made, each played to between two and four subjects [see supplementary methods in file S1]. Although, ideally, each individual would have heard a unique stimulus sequence, this was not possible due to the difficulty of obtaining high quality recordings of single individuals screaming in the two narrowly defined contexts. We thus tested individuals with a smaller set of high quality, realistic recordings. Using small stimulus sets can create problems with data interpretation, especially if subjects attend to peculiarities in the acoustic structure of stimuli instead of the conveyed meaning [24]. Field studies with primates have shown that individuals attend to the meaning of calls, not just their acoustic surface features [25], indicating that it is acceptable to use the same stimulus sequence on several individuals.

### Protocol

This study followed a counterbalanced within-subjects design, with six subjects receiving the incongruent condition first. Each subject had at least 7 days between trials (mean  = 24, SD = 10).

**Figure 2:**
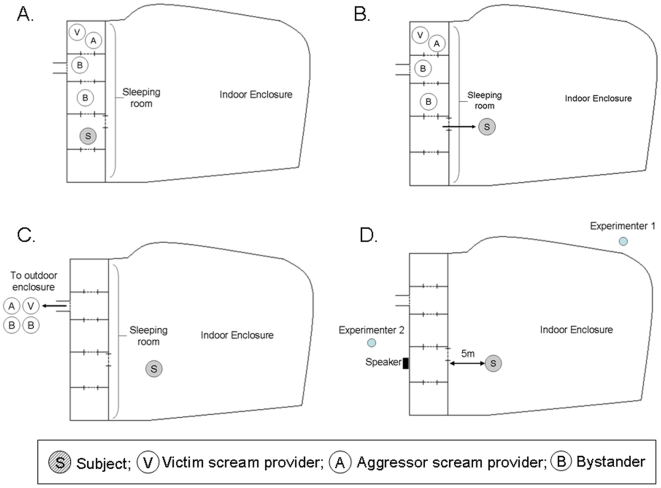
Schematic illustration of the experimental procedure for an incongruent trial. A. Initial position of all chimpanzees in sleeping room. B. Subject enters indoor enclosure. C. Call providers and bystanders enter outdoor enclosure. D. Subject positioned 5 m from sleeping room wall. Experimenter 1 films subject. Experimenter 2 broadcasts calls from sleeping room.

Each trial involved 6-7 different individuals: one subject, two or three call providers and two bystanders. The rest of the group were in the outdoor enclosure. We assumed that chimpanzees keep track of each other's whereabouts so we created spatially realistic constellations before broadcasting any stimuli. The subject was first separated from the others in the sleeping room, but he/she could still see 4 or 5 other individuals, including the call providers (fig. 2).

The subject was released into the indoor enclosure and usually approached the food (scattered muesli) placed 5 m from the sleeping room wall. The keeper then released all other individuals, including the call providers, into the outdoor enclosure, where they would not hear their own calls being broadcast. It was unavoidable that the subject would hear the hydraulic doors associated with releasing individuals outside, but due to the presence of the bystanders they could not know which of the initial 4 or 5 other individuals had been released. In order to create a realistic situation and give the impression that some unknown individuals remained in the sleeping room, the keeper then moved the internal doors and shouted in manner consistent with moving chimpanzees within the sleeping room. Experimenter 1, who filmed the subject, waited until the subject was sitting in the desired location for one minute. Experimenter 2 then broadcast the stimulus from the sleeping room. The subject's response to the playback was filmed, then after 5 minutes the keepers simulated the release of the call providers into the outdoor enclosure, by shouting and operating doors. The subject then rejoined the group in the outdoor enclosure.

### Measures

We coded the video footage frame-by-frame (25 frames/second) using Adobe Premiere Pro CS3. We measured (1) duration of looking towards the sleeping room in the minute before the playback; (2) duration of looking towards the sleeping room in the minute after the onset of the victim screams. As the congruent or incongruent nature of the stimulus sequences only emerged at the onset of the victim screams, we measured from this point (3) whether the subject approached the sleeping room doors in the minute after playback.

In order to ensure the videos had been accurately coded, 5/20 randomly chosen trials (25%) were recoded on the same three measures by an independent individual blind to the hypotheses and the trial type. We gained 100% agreement on whether the subject approached the sleeping room and a Pearson's correlation showed our duration measurements were also highly similar (Duration looking at sleeping room pre-playback, r = 0.992; Duration looking at sleeping room post-playback, r = 0.971), thus we had confidence the videos had been accurately coded.

Due to small sample sizes, we conducted two-tailed non parametric statistical tests, specifically Wilcoxon signed-rank and Binomial tests. In line with recommendations given by Mundry and Fischer [26] exact rather than asymptotic p-values are reported.

## Supporting Information

File S1 Details of acoustic analysis of stimuli and descriptive comparison to published acoustic structure of chimpanzee screams Details on the selection of stimulus sets for each subject.
(0.04 MB DOC)
