# Supplementary material for: Chimpanzees Extract Social Information from Agonistic Screams
Source: PLoS One. 2010 Jul 14;5(7):e11473. doi: 10.1371/journal.pone.0011473 (PMC2904366; doi:10.1371/journal.pone.0011473)
Supplement: File S1 — Details of acoustic analysis of stimuli and descriptive comparison to published acoustic structure of chimpanzee screams Details on the selection of stimulus sets for each subject. (0.04 MB DOC) [file pone.0011473.s001.doc]

**SUPPLEMENTARY METHODS**

*Acoustic analysis of the stimuli*

We performed acoustic analysis on the calls used as stimuli to ensure that victim and aggressor screams from this captive population were comparable to the distinct acoustic signals shown previously in a wild population (Slocombe and Zuberbühler, 2005). As we only used four aggressor screaming bouts and four victim screaming bouts as stimuli we were unable to perform statistical analyses on these calls, but we provide descriptive statistics to outline the structure of the calls and to allow for a descriptive comparison with the results from the wild population.

In order to facilitate direct comparisons with the analysis of the same calls from the wild, we performed nine of the same measures conducted on the wild calls. In line with Slocombe and Zuberbühler (2005) we used Raven Pro 1.3 to analyse the calls with the following settings: Hanning window function; filter bandwidth: 159 Hz; frequency resolution: 86.1 Hz; and grid time resolution: 0.113 ms.

In line with the previous research, most of the measures focused on the fundamental frequency (Fo) of each call. We performed the following measures on each call within each screaming bout used as stimuli in this experiment:

1. Duration of the call (s)
2. Frequency modulation (Hz): Highest frequency minus the lowest frequency in the Fo.
3. Peak frequency: Frequency at which maximum acoustic energy occurs in the Fo (Hz)
4. Relative transition—first quarter: Relative change in frequency during first quarter of the call divided by the total change in frequency occurring along the bottom edge of the Fo band, over the entire call (%).
5. Relative transition—second quarter: Relative change in frequency during second quarter of the call divided by the total change in frequency occurring along the bottom edge of the Fo band, over the entire call (%).
6. Relative transition—third quarter: Relative change in frequency during third quarter of the call divided by the total change in frequency occurring along the bottom edge of the Fo band, over the entire call (%)
7. Relative transition—fourth quarter: Relative change in frequency in the last quarter of the call divided by total change in frequency occurring along the bottom edge of the Fo band, over the entire call (%).
8. Absolute transition onset: Frequency of maximum energy at call onset minus frequency of maximum energy at call middle (Hz).
9. Absolute transition offset: Frequency of maximum energy at call middle minus frequency of maximum energy at call offset (Hz).

We created spectrogram slices (amplitude plotted against frequency) to determine the frequencies at which maximum acoustic energy occurred.

We took these nine measurements on each of the calls in each screaming bout. We then calculated a median value for each screaming bout for each of the nine measures. As we had small sample sizes the median values of aggressor screaming bouts (N=4) and victim screaming bouts (N=4) are shown in Table 1.

**Table 1. Median values of nine acoustic measurements for aggressor and victim screaming bouts.** Interquartile ranges are shown in parentheses.

|  | Aggressor (N=4) | Victim (N = 4) |
| --- | --- | --- |
| Duration (s) | 0.53 (0.09) | 0.53 (0.11) |
| Frequency modulation (Hz) | 984 (182) | 868 (121) |
| Peak Frequency (Hz) | 1324 (64) | 1288 (80) |
| Relative transition – 1^st^ quarter (%) | 43 (5) | 58 (7) |
| Relative transition – 2^nd^ quarter (%) | 25 (13) | 30 (5) |
| Relative transition – 3^rd^ quarter (%) | 65 (9) | 31 (7) |
| Relative transition – 4th quarter (%) | 61 (22) | 56 (11) |
| Absolute transition onset (Hz) | -177 (72) | -40 (74) |
| Absolute transition offset (Hz) | 329 (90) | 45 (83) |

The wild victim and aggressor screams did not significantly differ in peak frequency or absolute transition onset. Table 1 shows that peak frequency in the captive sample does not vary greatly. The absolute transition onset is smaller for victim screams in both populations, but this trend seems more exaggerated in the captive sample.

Slocombe and Zuberbühler (2005) found significant differences between victim and aggressor screams in six acoustic measures. The captive results mirror the pattern of results in four of the six measures. Aggressor screams have larger frequency modulations, smaller relative transitions in the 2^nd^ quarter, larger relative transitions in the 3rd quarter and larger absolute transition offsets (see Table 1). The two largest and most significant differences between victim and aggressor calls in the wild population were the relative transition in the 3rd quarter and absolute transition offset. Similarly the differences in these measures in the captive population also seem substantial.

The only measures that seem to differ between the wild and captive populations are duration and the relative transition in the 4^th^ quarter. In the wild aggressor screams were significantly shorter than victim calls, but the captive population screams were the same median duration. In the wild the relative transition in the 4^th^ quarter was significantly larger for the victim screams, where as the captive calls show a small difference in the opposite direction.

To summarise the captive victim and aggressor screams share most of the acoustic features of victim and aggressor calls described in a wild population. The key differences in victim and aggressor screams in both populations concern the shape of the call. Victim screams are relatively flat, whilst aggressor screams have a distinctive decrease in frequency in the second part of the call.

*Relationships between subjects and scream providers*

In order to determine which stimulus each subject should hear, we used the 2007 observational data from WKPRC (all occurrence sampling of agonistic interactions, collected in 2 hour periods 4-5 times/week) on support during agonistic interactions. We gave the subject the stimulus that was likely to be most relevant i.e. the one that contained the most individuals they habitually supported in agonistic contexts. Support during agonistic interactions included the following behaviours: aggression towards aggressor in terms of contact, chasing or threatening or support for the victim in terms of approaching and embracing or grooming.

One female, Dorien, gave an exceptionally strong response to her first congruent trial, which appeared an outlier in comparison to her response to the incongruent stimulus and the responses of the other 9 chimpanzees to congruent stimuli (more than 2.5 std deviations from the mean). We thus examined the long term support data to see if there was a reason for this. On closer examination, the congruent stimulus contained the three individuals Dorien most commonly supported (32/34 (94%) events where she gave support in 2007 were to help these three individuals). In contrast no other subject had a strong supporting relationship with all of the congruent stimulus providers. Five subjects had tendencies to support 2/3 of the congruent stimulus providers (accounting for a mean of 62% total support given, SD = 18), three individuals had tendencies to support 1/3 of the congruent stimulus providers (accounting for a mean of 39% total support given, SD = 19) and one subject had weak tendencies to support 3/3 of the congruent stimulus providers, but this only accounted for 56% of her total support given.

We thus retested Dorien on a different stimulus set in 2008, for which she only had a strong tendency to support one individual providing the congruent stimuli (accounting for 70% of her total support). The magnitude of Dorien’s responses to both conditions of this new stimulus set were in line with all other subjects. We thus report the 2008 results rather than the 2007 results for this individual in the results section.

*Supplementary references*

Slocombe, K. E. and Zuberbühler, K. (2005) Agonistic screams in wild chimpanzees vary as a function of social role, *J. Comp. Psych.*, **119(1),** 67-77
